# Do natural experiments have an important future in the study of mental disorders?

**DOI:** 10.1017/S0033291718003896

**Published:** 2019-01-04

**Authors:** Anita Thapar, Michael Rutter

**Affiliations:** 1 Child & Adolescent Psychiatry Section, Division of Psychological Medicine and Clinical Neurosciences, MRC Centre for Neuropsychiatric Genetics and Genomics, Cardiff University School of Medicine, Hadyn Ellis Building, Maindy Road, Cathays, Cardiff, CF24 4HQ, UK; 2 MRC SGDP Centre, Institute of Psychiatry, Psychology and Neuroscience, King's College, London, UK

**Keywords:** Causal, causes, environment, epidemiology, genetics, natural experiments, psychiatric disorders

## Abstract

There is an enormous interest in identifying the causes of psychiatric disorders but there are considerable challenges in identifying which risks are genuinely causal. Traditionally risk factors have been inferred from observational designs. However, association with psychiatric outcome does not equate to causation. There are a number of threats that clinicians and researchers face in making causal inferences from traditional observational designs because adversities or exposures are not randomly allocated to individuals. Natural experiments provide an alternative strategy to randomized controlled trials as they take advantage of situations whereby links between exposure and other variables are separated by naturally occurring events or situations. In this review, we describe a growing range of different types of natural experiment and highlight that there is a greater confidence about findings where there is a convergence of findings across different designs. For example, exposure to hostile parenting is consistently found to be associated with conduct problems using different natural experiment designs providing support for this being a causal risk factor. Different genetically informative designs have repeatedly found that exposure to negative life events and being bullied are linked to later depression. However, for exposure to prenatal cigarette smoking, while findings from natural experiment designs are consistent with a causal effect on offspring lower birth weight, they do not support the hypothesis that intra-uterine cigarette smoking has a causal effect on attention-deficit/hyperactivity disorder and conduct problems and emerging findings highlight caution about inferring causal effects on bipolar disorder and schizophrenia.

## Introduction

Psychiatric disorders have a complex etiology; influenced by multiple genetic as well as environmental risk factors. Although most are heritable, in the shorter term, environmental factors are more tractable to modification. If environmental exposures are causal, then their modification should lead to improvements in population health or the psychiatric disorder being treated (see Table 1: meaning of key terms). Thus it is important to assess which of our selected environmental exposures are genuinely causal – but this is challenging.

**Table 1.** Key terms and what they mean

Risk	Probability of an outcome in a given population
Risk factor	A measurable exposure or agent that precedes the outcome and is statistically associated with it
Correlate	Meets criteria for risk factor but is measured at the same time or after (thus not known to precede outcome)
Causal risk factor	A risk factor that changes risk of outcome when altered
Selection bias	Systematic differences between baseline characteristics of the groups compared (e.g. those participating in study *v.* those not)
Allocation bias	Systematic difference between how participants are allocated to an intervention or exposure group (e.g. in an RCT)
Negative control (could be exposure or outcome)	This could be an exposure (e.g. intra-uterine exposure to paternal influenza virus) or outcome that is thought to be subject to similar confounding as the exposure of interest (e.g. intra-uterine exposure to maternal influenza virus) but that does not affect the outcome (e.g. schizophrenia) This method can be used to identify and deal with unmeasured confounding and other biases, e.g. selection bias

Kraemer *et al*., 1997; Sedgwick, 2013; Arnold and Ercumen, 2016.

Traditionally many of the exposures that we believe to be risk factors for psychiatric disorder have been implicated through observational designs. These infer causation from observations of association. However, association is not causation. Threats to causal inference include reverse causation, confounding, and selection bias (Rutter, 2007; Thapar and Rutter, 2015). For example, has the supposed risk factor of family discord arisen as the result of the individual's psychiatric disorder – reverse causation? Has the common factor of social disadvantage contributed to both the outcome of psychiatric disorder and family discord – confounding? Does cannabis have a causal risk effect on schizophrenia or is it that those with a higher propensity to develop schizophrenia are more likely to use cannabis – selection bias?

These are important threats because if they lead to misleading and inconsistent conclusions, this confuses clinicians, researchers, the general public, and patients. At worst it leads to wasted resources. The challenges in inferring causality are not just restricted to psychiatry. For example, observational studies suggested vitamin E had a protective effect on cardiovascular disease until randomized controlled trials (RCTs) suggested that this was not the case (Eidelman *et al*., 2004). RCTs are often considered as the ‘gold standard’ for assessing causal effects. However, given that RCTs of many environmental exposures relevant to psychopathology are not going to be feasible or ethical, what is the alternative?

‘Natural experiments’ provide an alternative strategy. We refer to designs that take advantage of situations whereby links between the exposure and other variables are separated by naturally occurring events or situations. Unlike RCTs, the manipulation is not undertaken by the researcher. Some involve the design and others the statistical methods.

In this review, we will consider some types of natural experiments and describe how they have been applied in the field of psychiatry. The aim is not to provide an exhaustive account of different methods but rather to focus on the principles, design, and limitations. There are a number of other methods that in the interests of space will not be covered in this review but are discussed elsewhere (Rutter and Thapar, 2018). Although there are other reviews (e.g. Pingault *et al*., 2018), we aim to describe a broad range of designs and will provide examples of findings that would be relevant to a clinician.

There is a growing trend toward viewing causal inference as a single approach based on considering what would have occurred if an individual had not been exposed to the risk? [see Krieger and Davey Smith (2016) for an excellent discussion]. However, we agree with Krieger and Davey Smith (2016) for taking a broader view; one that emphasizes convergence or ‘triangulation’ of findings across diverse types of designs that have different types of biases and assumptions. When the same finding is observed using different approaches, it provides greater confidence in inferring causality especially when such studies are conducted in different populations.

## Genetically informative designs that remove familial and genetic confounding

Many of the most important risk factors for psychopathology, such as life events and inter-personal discord, are person-dependent; they are not randomly allocated. Thus, it is unsurprising that decades of research have shown that many types of adversities run in families and are heritable (e.g. McGuffin *et al*., 1988; Plomin, 2018). This raises the possibility that an association between exposure and psychiatric outcome could arise through familial or genetic confounding (Thapar and Rutter, 2009, 2015).

It is for this reason that genetically informative designs such as twin studies have been invaluable for testing whether links between environmental exposures and psychopathology remain associated once genetic or familial confounds are taken into account.

Some designs, such as the discordant sib pair and *in vitro* fertilization (IVF) design (Thapar *et al*., 2007), enable removal of genetic or familial confounds for prenatal exposures. For example, prenatal exposure to cigarette smoke has been linked with later risk for offspring attention-deficit/hyperactivity disorder (ADHD), conduct disorder, bipolar disorder, and schizophrenia. The effects could potentially be causal; for example, mediated by effects of nicotine on the developing brain. However, unmeasured confounds and selection biases are a concern, meaning that natural experiment designs have proved very useful here (Quinn *et al*., 2017; Rice *et al*., 2018).

Twin and adoption studies are not able to separate genetic confounds for prenatal exposures. That is because twins share their prenatal exposures and varying degrees of genetic liability and for adopted offspring, it is their biological mother who provides both the prenatal environmental and half of their genetic makeup. However, such designs are well-suited for assessing post-natal exposures. Some designs such as the children-of-twins design (D'Onofrio *et al*., 2003) and adoption designs are especially well-suited for examining cross-generational environmental as well as genetic transmission (see Table 2).

**Table 2.** Genetically informative designs and what they can be used to assess

	Prenatal exposures	Postnatal exposures	Cross-generational transmission
IVF design	+	+	+
Maternal *v.* paternal exposure	+		
Discordant sib pair design	+	+	
Twin design		+	
MZ twin discordance		+	
Children of twin design	+	+	+
Adoption design		+	+

### Maternal *v.* paternal exposure during pregnancy

**Fig. 1.**
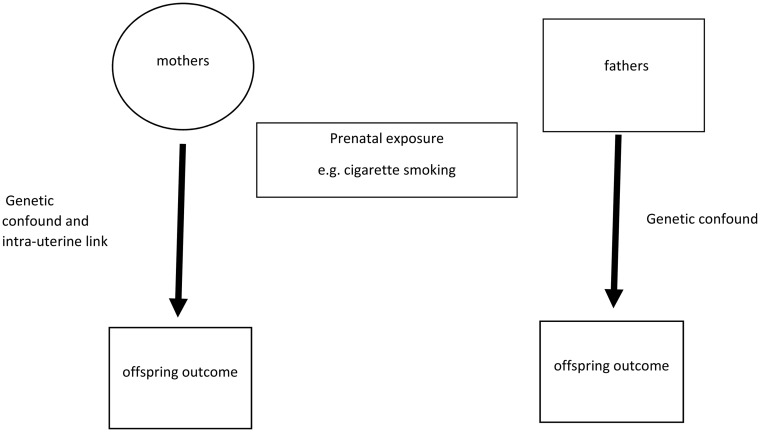
Maternal *v.* paternal exposure.

One method that has been used to disaggregate intra-uterine and genetic or house-hold/familial-level influences involves testing associations between maternal *v.* paternal exposures during pregnancy and offspring outcomes (Fig. 1). If the link is mediated by an intra-uterine effect, a stronger association would be expected for the maternal exposure. For example, in a UK population-birth cohort ALSPAC, strong associations were observed between maternal smoking in pregnancy and shorter birth length (Howe *et al*., 2016) and lower birth weight in offspring (Langley *et al*., 2012) that were not observed when exposure to paternal smoking was examined (see Table 1; this is an example of a negative control exposure). However, in this same cohort, associations between exposure to smoking in pregnancy and ADHD were as strong for maternal exposures as they were for paternal exposures even in the case of mothers who did not smoke. These results held when the contribution of additional passive smoking was considered. There are limitations to this design including the fact that parents will show similarities in exposures due to genetic (assortative mating) and social reasons and it is restricted to the sorts of exposures that both parents could feasibly experience in pregnancy.

### Discordant sibling pair design

Full biologic siblings share on average 50% of their genome. Thus differences between them can be used to assess family-level confounds that include genetic and shared environmental contributions.

(i) Prenatal exposures. As they share the same mother, they become of special interest when they have been differentially exposed to prenatal factors. For example, taking the example of maternal smoking in pregnancy and ADHD, eight studies of discordant sibling pairs have now found that the siblings who were unexposed to smoking in utero showed elevated levels of ADHD (Rice *et al*., 2018). Similar findings were observed for conduct problems. Birth weight provided the negative control as the studies that examined this outcome found that the association with cigarette smoking remained strong. A recent, large discordant sibling study also failed to find support for a causal effect of exposure to prenatal smoking on severe mental illness (bipolar disorder and schizophrenia) suggesting the contribution of family-level confounders to previously observed associations (Quinn *et al*., 2017). There are many limitations to this design that have been described elsewhere. These include the issue of selection as mothers are behaving differently in different pregnancies. For example, the sample consists of a group of mothers who are able to quit smoking in one pregnancy but not the other. Also, there is the problem that siblings will be born at different times and thus will be exposed to different family-level and population-level risks.

(ii) Assessing later adversities using a sibling pair design and its extension, the co-relative study. The discordant sibling pair design and its extension involving pairs of relatives from the same generation such as half-siblings and cousins have also been used to assess causal links between adolescent and adult exposures and psychiatric disorders. For example, the observed association between cannabis use and schizophrenia has been well-established. However, the causal relationship could be subject to question given that those who are at elevated familial or genetic liability or with prodromal symptoms could be more likely to use cannabis (confounding, selection bias, and reverse causation). In one large, Swedish study, the authors used an extended sibling pair design to investigate the causal relationship between cannabis and schizophrenia (Giordano *et al*., 2015). The association was much attenuated once familial confounding was taken into account; the effect size also was diminished when potential prodromal effects were considered that was assessed by increasing the temporal delay between cannabis abuse and admission for schizophrenia (odds ratio 1.67). The findings suggested that there is a likely causal link between cannabis use and schizophrenia for some but that the effect size is not as strong as previously reported because of the contribution of familial confounding and reverse causation.

### IVF design

**Fig. 2.**
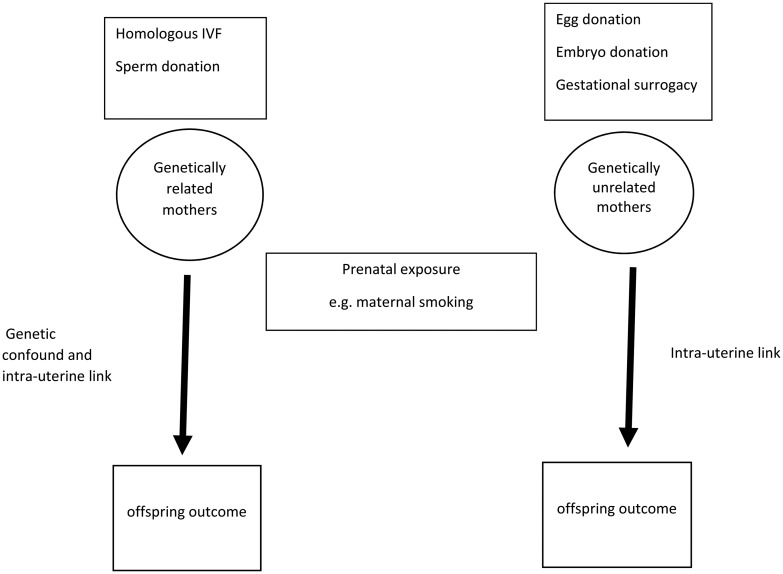
*In-vitro* fertilisation design.

An alternative design that enables separation of prenatal exposures from genetic ones is based on individuals who have been conceived through assisted reproductive technologies. Some of these individuals are genetically related to the woman who undergoes the pregnancy and others are genetically unrelated (see Fig. 2). If a prenatal exposure has causal effects, then association with the offspring outcome should be observed regardless of whether mother–offspring dyads are genetically related or unrelated. That was the case for maternal smoking in pregnancy and lower birth weight (Thapar *et al*., 2009) and also for associations between maternal reports of stress in pregnancy and lower birth weight and preterm birth (Rice *et al*., 2010).

However, for association between maternal smoking in pregnancy and a trait measure of ADHD in offspring (Thapar *et al*., 2009) as well as conduct problems (Rice *et al*., 2009), association was only observed in genetically related mother–offspring dyads not in the unrelated pairs, suggesting genetic confounding. The finding converges with those from the maternal *v.* paternal exposure and discordant sibling pair designs. Interestingly, the magnitude of association in the related pairs was similar to that observed in other observational studies and including measured confounders of the sort including in observational designs, such as parental psychopathology, social class did not remove the genetic confound.

That is, findings from this and other studies suggest that residual confounds remain a problem for observational studies and that including multiple confounders is not a substitute for an informative design.

The IVF design (Thapar *et al*., 2007) has also been used to assess inter-generational transmission of psychopathology and to examine post-natal adversity. For example, using this approach, depression symptoms were found to be environmentally transmitted and environmental links were observed between hostile parenting and antisocial behavior in offspring (Harold *et al*., 2011).

The IVF design does have a number of limitations however. These include the representativeness of the families who have undergone IVF treatment and the low prevalence of certain types of risk factors (e.g. maternal smoking in pregnancy).

### Twin designs

Twin designs utilize the fact that monozygotic (MZ) twins share on average 100% of their genes (DNA sequence) and dizygotic (DZ) twins share on average 50% of their genome.

The twin design allows variation in any given measure to be partitioned into genetic and environmental variance. Where both exposure and psychiatric outcome are assessed, ideally longitudinally to avoid the problem of reverse causation, the association between exposure (e.g. life events) and outcome (e.g. depression) can be decomposed into genetic and environmental components. As the genetic covariance between exposure and outcome is explicitly modelled, essentially the genetic confound is removed. Here, the investigator is interested in whether there is an environmental link that remains between the exposure and outcome. This design has been invaluable in demonstrating a number of potentially causal environmental risk factors for psychopathology.

For example, family and twin studies of depression in childhood, adolescence, and adult life have observed a familial and genetic contribution to life events, mainly those that are person-dependent (e.g. losing a job) rather than ones that are independent (death of a relative), as well as to depression (McGuffin *et al*., 1988; Plomin, 2018).

Twin studies that have investigated the link between life events and depression suggest that the association between independent life events and depression appears to be mainly or entirely environmental; that is consistent with a causal explanation (Kendler *et al*., 1999). For dependent life events, there is a stronger genetic contribution to the link with depression. This seems to be partly explained by self-selection into risk exposure by those predisposed to depression (Kendler *et al*., 1999) and becomes more prominent from adolescence onwards (Rice *et al*., 2003).

Another example is the link between harsh parenting and antisocial behavior in children. One twin study found that the association with corporal punishment was primarily explained by genetic factors (Jaffee *et al*., 2004). This could arise, for example, through parental response to the child's behavior which is genetically influenced. However, the findings for physical abuse were different. Here, the link with antisocial behavior was environmentally mediated, and consistent with a causal explanation.

Twin designs, their uses, strengths, and limitations have been described in detail elsewhere (State and Thapar, 2015). When genetic contributions are identified through bivariate twin analyses that we have described, it can index selection bias and potential threats to causal inference. However even with longitudinal twin designs, an environmentally mediated link does not prove a causal link between an exposure and outcome as there could be alternative pathways that explain the association including measurement artifacts.

### Discordant MZ twin pairs

This design utilizes the fact that MZ twins are considered to share 100% of their genes and means that differences in their phenotype are attributed to non-genetic contributions that include non-shared environment as well as measurement error and stochastic effects. The approach involves assessing whether MZ twins who are differentially exposed to a stressor or adversity (e.g. discordant for victimization) show differences in a given outcome (e.g. depression).

For example, in the UK E-risk twin study of 7–10 years old, 110 MZ twin pairs who were discordant for bullying victimization were assessed (Arseneault *et al*., 2008). The co-twins who were bullied showed higher internalizing (anxiety/depression) symptom scores than those who were not exposed to bullying. A more recent US longitudinal twin study also investigated 145 MZ twins who were discordant for bullying victimization in childhood (Silberg *et al*., 2016). Although being bullied showed a genetic link with social anxiety; there were also environmental links with social anxiety, separation anxiety, and young adult suicidal ideation. The findings from both of these studies are consistent with a causal effect of bullying victimization on emotional/anxiety symptoms and are important given the interest in reports from longitudinal observational designs.

In another longitudinal MZ discordant twin study (Caspi *et al*., 2004), Caspi *et al*. assessed maternal hostility and warmth. This was achieved by conducting independent ratings from a recorded 5 min speech sample from the mother when talking about the child (expressed emotion EE). Maternal expressed emotion was found to be environmentally associated with later teacher-reported behavioral problems.

Although the MZ discordant pair design is useful because it controls for genetic confounding there are some drawbacks. For example, we now know that MZ twins are not *per se* 100% genetically identical, for example, through non-inherited genetic differences. Also discordant MZ twin pairs could be considered as atypical and rare especially for very highly heritable disorders such as autism or ADHD or schizophrenia. The exposure could be behaving as a proxy for some other risk factor that impacted on one twin and not the other.

### Children of Twins design and extensions

The Children of Twins (CoT) design allow investigation of cross-generational links between parent and offspring psychopathology or parentally provided exposures and offspring outcomes. It takes advantage of the fact that the offspring of MZ and DZ twins are socially cousins (DZ twins are also genetically cousins) but the MZ twin offspring are genetically half siblings.

This type of design, for example, has been used to assess the cross-generational transmission of depression. In an Australian study of twins, their spouse, and offspring, environmental factors were found to explain the link between parents and offspring depression even when accounting for depression in spouses (Singh *et al*., 2011). Similar findings had been found in an earlier US study (Silberg *et al*., 2010). Another CoT study from Sweden found that depression symptoms in parents showed concurrent environmental but not genetic links with offspring internalizing symptoms (McAdams *et al*., 2015). The findings accord with those from the IVF study (Harold *et al*., 2011). A more recent Swedish CoT design observed only environmental transmission between parents and offspring for anxiety and neuroticism; again with no genetic contribution (Eley *et al*., 2015).

These findings might appear puzzling in that while it is important to observe environmental transmission of depression and anxiety, there are no genetic contributions observed for either and this is inconsistent with twin studies (Sullivan *et al*., 2000). Twin studies observe modest heritability for depression. One difficulty for cross-generational investigations is the assumption that the same genetic influences contribute across development when that is unlikely (e.g. Power *et al*., 2017; Riglin *et al*., 2017). Another issue is that twin study heritability estimates capture passive gene–environment correlation effects that would be reduced in CoT studies and eliminated in the IVF design.

The CoT design has also been used to assess postnatal adversities. One such study (Lynch *et al*., 2006) found that harsh physical punishment remained associated with childhood behavioral problems even when genetic factors had been allowed for. These findings are in keeping with the twin study findings and taken together are consistent with harsh parenting having a causal effect on childhood antisocial behavior.

### Adoption studies

Adoption studies allow genetic and prenatal influences to be separated from post-adoption experiences. They provide a powerful method for assessing the contribution of rearing influences because these are known to be affected by with genetically influenced parental attributes. Ordinarily these biological parental characteristics would in turn be correlated with child characteristics including psychopathology thereby introducing a potential genetic confound. The advantage of adoption studies is that they remove this confound, the so-called passive gene–environment correlation because the genotypes of the parents who are rearing the children are independent of the child's genotypes.

There are several examples where adoption studies have been able to demonstrate the contribution of the rearing environment. For example, a study of adopted away children showed that negative parenting provided by the adoptive parent was associated with their adoptive child's antisocial behavior (Ge *et al*., 1996). The adoptive parent's negative parenting was also associated with substance abuse/dependency or antisocial personality in the child's biological parents; that association appeared to be mediated via the child's behavior. Overall the findings suggested causal effects of negative parenting on children's antisocial behavior but also showed that the children's genetically influenced antisocial behavior in turn affected the parenting of the adoptive parents. The observation that negative parenting has a causal effect on offspring antisocial behavior converges with the findings from twin studies showing a convergence of findings from different designs.

A more recent example is provided by a Swedish large-scale adoption study cross-generational study (Kendler *et al*., 2018). The authors were able to assess the contribution of genetic and rearing influences to parent–offspring resemblance for treated major depressive disorder. They found that both genetic and rearing influences contributed equally to parent–offspring resemblance in major depressive disorder. The adoptive families enabled the authors to further show that genetic and rearing influences acted additively rather than having an interactive effect. The authors highlighted that there had been four previous adoption studies of depression; although genetic contributions had previously been observed, only one had observed an environmental contribution to depression. However, now there have been two adoption studies that have showed an environmental contribution to inter-generational transmission of depression. Also the same findings have been observed in three children of twin designs and in the IVF design, although here some of these find environmental contributions only with no genetic transmission.

Overall the findings from different genetically informative studies of depression are converging on the suggestion that environmental/social factors contribute to the cross-generational transmission of depression. That of course has important clinical treatment and prevention implications.

## Designs involving the introduction or removal of risks to a population: potentially removing selection or allocation bias

Given a serious challenge to causal inference is selection or allocation bias, a number of studies have taken advantage of situations where risks have been introduced to or removed from an entire population.

### Universal introduction of risk

Here, the best known studies are the Dutch Hunger Winter (Susser *et al*., 1996) and Chinese famine studies (St Clair *et al*., 2005) that examined the consequences of intra-uterine exposure to famine. These studies focused on populations that were exposed to universal time-limited famines that affected some individuals during the intra-uterine period. Exposed individuals in both studies showed around a twofold elevated risk of schizophrenia as well as congenital anomalies of the central nervous system. As there was no evidence for selection for exposure to either of these famines, the findings suggest that extreme nutritional deficiency in early pregnancy likely has a causal risk effect for schizophrenia. However, the conditions in both of these studies was extreme and atypical so whether the findings have relevance for the etiology of schizophrenia as a whole is unknown.

### Universal removal of risk

In this design, the strength is that it again removes selection or allocation bias whereby the person or some external agent influences the removal of risk. One good example is provided by the Great Smoky Mountains Study that is a longitudinal epidemiological study. During the course of this study of over 1000 children, a casino opened on a Native American reserve and provided a substantial increase to the family income for around a quarter of the original sample. The investigators were able to examine data before and after this happened. They showed that the relief of poverty led to decreased levels of oppositional defiant disorder and conduct disorder but not anxiety or depression (Costello *et al*., 2003). The effects appeared to be mediated via altered parenting that included increased levels of supervision and parental time. Later follow-up showed that family income supplementation provided in childhood continued to be associated with lower rates of psychiatric problems including alcohol and cannabis abuse, lower rates of convictions for minor offenses, and higher levels of education. There were no links with later behavioral disorders or depression or other drug use (Costello *et al*., 2010).

### Interrupted time series

This design takes advantage of multiple waves of data that have been collected before and after the introduction or removal of the putative causal variable. This could be used to assess the impact of a policy or a naturally occurring event.

For example, after the introduction of UK legislation to reduce paracetamol package sizes, there was an observed drop in deaths from paracetamol overdoses (Hawton *et al*., 2013).

Another example comes from a study of gang membership that is known to be associated with higher rates of delinquency (Thornberry *et al*., 1993). However, it is not known whether that is due to selection effects with those having a propensity to be delinquent choosing to be in a gang or whether it is the causal social effects of being in a gang. Thornberry *et al*. (2002) found as might be expected important selection effects; boys who joined gangs were more delinquent than those who did not. However, they also showed that once boys left the gang, their rates of delinquency dropped off though not back to the level they were prior to joining the gang. This observation suggested that gang membership had additional social influences on delinquency. However, reverse causation and unmeasured confounders are possible contributors because we do not know what affected the boys’ decisions to leave the gang.

### Changes in policy

If these are applied to a whole nation and data are available before and after the introduction of the policy, then this can provide a useful natural experiment situation. One study in Sweden (Nilsson, 2008) focused on the effects of prenatal alcohol exposure in two regions that were subjected to an experimental policy change in alcohol sales. The intention was to shift the population away from drinking spirits to consuming drinks with a lower alcohol content. However, it inadvertently resulted in very marked increases in the consumption of strong beer especially amongst teenagers. The experimental policy started in 1967 but was terminated abruptly in mid-1968 once it was realized that alcohol consumption had increased. Using registry data, the researchers were able to assess a cohort of children who had been *in utero* during the exposed period. As the policy was time and geographically limited, the exposed cohort could be compared with unexposed cohorts in adjacent geographic regions and in adjacent time-unexposed cohorts. At around 30 years of age, the exposed group showed greatly reduced educational achievements, lower earnings, and greater welfare dependency than those born to the unexposed cohorts. The effects were strongest in males, those exposed for the longest in intrauterine life and those born in younger mothers. The results suggest that prenatal exposure to alcohol likely had intrauterine risk effects on offspring. However, the problem with this sort of policy study is that the results are obtained from analyses at a group rather than individual level.

## Radical change in environment: adoption following profound institutional deprivation

One good example of a natural experiment was provided by the English and Romanian Adoptees Study that involved a very radical change in early environment. This is a longitudinal study of individuals who were exposed to institutional care and extreme privation from early infancy. The possibilities of selection bias and reverse causation were essentially removed because the children were admitted very early and virtually no children left care until the government regime fell in 1989. These children subsequently were exposed to a radical change in rearing environment after they were adopted into relatively advantaged homes in the UK. The findings from this study showed that although there was some recovery, early institutional care of the type experienced by these children for more than 6 months resulted in difficulties that persisted to adulthood including autistic-type symptoms, ADHD-like problems, disinhibited social engagement, and emotional symptoms but not cognitive impairment (Sonuga-Barke *et al*., 2017).

As is the case for some of the other natural experiments, such as the famine studies, although selection bias is removed, the question is whether the findings apply to less severe and more common forms of deprivation.

## Using instrumental variables as a statistical method to deal with unmeasured confounding

An instrumental variable is a measured variable that is associated with the exposure of interest but that is not associated with the same selection effects and confounds. If the exposure has a genuinely causal risk effect on the outcome, then we would expect the instrumental variable also to be associated with the outcome. Early use and misuse of alcohol have been considered as potential causal risks or exposures for the later outcomes of alcohol dependence and misuse in adult life. Early puberty has been used as an instrumental variable for early use and misuse of alcohol because it is strongly associated with these exposures yet is not subject to the same selection biases or confounds.

Three studies have found that while early alcohol use and misuse in adolescence is associated with later alcohol problems, early puberty does not predict alcohol problems (Stattin and Magnusson, 1990; Caspi and Moffitt, 1991; Pulkkinen *et al*., 2006).

These findings suggest that early alcohol use is likely an early manifestation of later alcohol problems rather than a cause of it.

## Mendelian randomization: a special type of instrumental variable

**Fig. 3.**
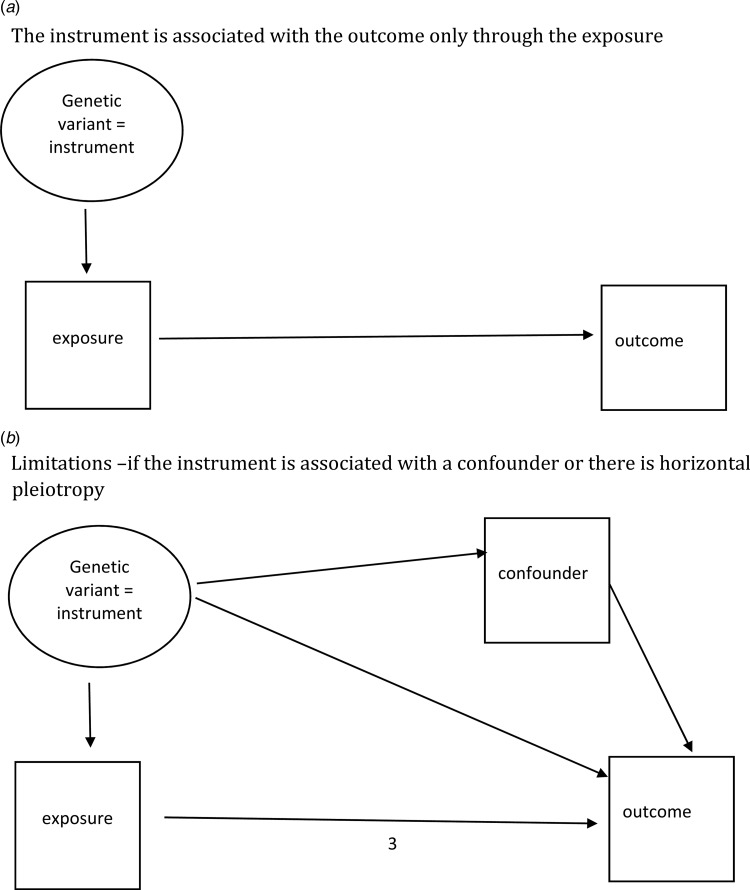
Mendelian randomization. (*a*) The instrument is associated with the outcome only through the exposure. (*b*) Limitations – if the instrument is associated with a confounder or there is a horizontal pleiotropy.

Mendelian randomization (MR) utilizes the random assortment of parental genotypes to offspring during meiosis. Here a genetic variant that is robustly associated with the exposure is used as the instrumental variable and provided certain assumptions are met should provide a means of controlling for confounding and reverse causation (see Fig. 3). As more genetic variants are being identified through genome-wide association studies, there is a growing interest in using MR to test causal hypotheses and many methodological extensions of this approach (Davey Smith and Hemani, 2014). One approach called two-sample MR takes advantage of already published large genome-wide association studies. It uses genetic variants for exposures [e.g. C reactive protein (CRP)] as instrumental variables and another set of genetic variants from another independent GWAS for the outcomes variants (e.g. cardiovascular disease). MR has been used most successfully in relation to cardiovascular disease. For example, MR has been used to show that CRP does not have a causal risk effect on cardiovascular disease (C Reactive Protein Coronary Heart Disease Genetics Collaboration (CCGC) *et al*., 2011). More recently, MR has started to be used in psychiatry; for example, a recent study observed body mass index effects on depression but not the reverse (Nagel *et al*., 2018). MR is challenging because of its assumptions. For example, there is a need for genetic variants that have a strong and robust association with the exposure in question, although there are methods that allow for combining multiple genome-wide significant variants. Also if the genetic variant (instrument) has pleiotropic effects, and that is often the case, or influences a confounder or affects the outcome via another mechanism other than via the exposure, then that poses problems. There are methods for assessing pleiotropy and again, like all the methods we have discussed, MR findings on their own need to be interpreted with caution. However, when findings converge with other designs, they can be helpful in inferring causation. They are also a helpful alternative to RCTs.

## Conclusions

It is crucial that genuinely causal influences on psychopathology are identified if interventions and policies are going to be effective. In recent years, findings relevant to psychiatry have emerged from different natural experiment designs and some are consistent across different designs; this strengthens causal inference. For example, hostile parenting affects antisocial behavior and RCTs uphold this causal inference. Genetically informative studies converge in favor of life events and victimization being environmentally linked with depression and environmental cross-generational transmission for depression. However, although smoking cessation programs for pregnant women are clearly a priority as cigarette smoke is detrimental to offspring physical health, the natural experiment designs suggest these will not be a useful means for preventing ADHD or antisocial behavior. So do natural experiments have an important future in the study of mental disorders? The answer is a firm yes.
